# Orthorexia and perfectionism in medical students in Tunisia

**DOI:** 10.1192/j.eurpsy.2024.1170

**Published:** 2024-08-27

**Authors:** A. Mellouli, S. Ellouze, N. Boussaid, M. Turki, A. Zribi, N. Halouani, J. Aloulou

**Affiliations:** Psychiatry B, Hedi Chaker University Hospital, Sfax, Tunisia

## Abstract

**Introduction:**

Orthorexia is defined as a dependence on healthy food or an obsession to consume healthy food. One area deemed influential upon disordered eating and dietary intake is perfectionism.

**Objectives:**

To investigate the relationship between orthorexia and perfectionism in medical students.

**Methods:**

We conducted a cross-sectional, descriptive, and analytical study in the faculty of medicine of Sfax (Tunisia), between February and April 2023. A self-reported questionnaire was distributed to students via social media. We used ORTO-15 for the assessment of orthorexia, and the Big Three Perfectionism Scale (BTPS) to assess perfectionism.

**Results:**

The research has enrolled 220 students. Their mean age was 21.40±1.68 years, with female predominance (70%). The ORTO-15 mean total score was 36.88±6.76. A total of 60% of participants had an orthorexic behavior. The BTPS mean total score was 45.52±12.45 with a mean score of 13.25±4.35 for rigid perfectionism, 18.31±6.37 for self-critical perfectionism, and 13.99±5.47 for narcissistic perfectionism.

Students with orthorexic behavior had significantly higher scores of perfectionism (p= 0.048).

**Conclusions:**

Our study has drawn a significant association between orthorexia and perfectionism among medical students. This result suggests that students experiencing highly critical and judgemental beliefs associated with perfectionism are more susceptible to orthorexic eating behaviors.

**Disclosure of Interest:**

None Declared

